# “Groupitizing”: a strategy for numerosity estimation

**DOI:** 10.1038/s41598-020-68111-1

**Published:** 2020-08-10

**Authors:** Giovanni Anobile, Elisa Castaldi, Paula A. Maldonado Moscoso, David C. Burr, Roberto Arrighi

**Affiliations:** 1 Department of Neuroscience, Psychology, Pharmacology and Child Health, University of Florence, Florence, Italy; 2 Department of Translational Research and New technologies in Medicine and Surgery, University of Pisa, Pisa, Italy; 3 Institute of Neuroscience, National Research Council, Pisa, Italy

**Keywords:** Human behaviour, Cognitive neuroscience

## Abstract

Previous work has shown that when arrays of objects are grouped within clusters, participants can enumerate their numerosity more rapidly than when objects are randomly scattered, a phenomenon termed “groupitizing”. Importantly, the magnitude of the grouping advantage correlates with math abilities in children. Here we show that sensory precision of numerosity estimation is also improved when grouping cues are available, by up to 20%. The grouping can be induced by color and/or spatial proximity, and occurs in temporal sequences as well as spatial arrays. The improvement is strongest for participants with the highest thresholds in the random, ungrouped conditions. Taken together with previous research, our data suggest that measurements correlations between numerosity estimation and formal math skills may be driven by grouping strategies, which require a minimal level of basic arithmetic.

## Introduction

When counting is not possible, humans and other animals can rapidly estimate the number of items in any scene to achieve an approximate assessment of their numerosity. The system sustaining this ability is often termed the *Approximate Number System* (ANS) and, like all sensory systems, is error-prone, with errors increasing proportionally with numerosity, obeying Weber Law^1–4^. Importantly, many studies have found a correlation between ANS precision (measured by Weber fraction or Coefficient of variation) and child math abilities, with lower precision associated with poorer performance in math^5–8^. Children with dyscalculia, a neurodevelopmental disorder affecting mathematical and numerical learning, often show higher Weber fractions compared to typically developing children^6,9,10^. Based on this evidence it has been proposed that the ANS may constitute a foundational non-symbolic system on which the language-based mathematical system could subsequently be built^6^.

Jevons^11^ first reported that estimates of numerosities less than four are fast and error-free, subsequently termed *subitizing* by Kaufman and Lord^12^. Subitizing is robust, and occurs for both sequential and simultaneous stimuli, in all sensory modalities^3,13–16^. Subitizing is highly dependent on attention^17–24^, and seems to work in parallel with the estimation system, boosting performance at low numerosities.

More recently, Starkey and McCandliss^25^ suggested that subitizing mechanisms may also come into play for higher numerosities, a process they term "*groupitizing*". This is very much like George Miller’s well-known notion of “chunking”, where complex sets of information such as long telephone numbers can be more easily recalled if parsed into three or four smaller “chunks”. Starkey et al.^25^ measured counting speed of spatially clustered arrays in school-age children, and found that clustering, or grouping, increased performance. Crucially, both the number of clusters and the number of elements within each cluster was limited to the subitizing range (e.g. 7 = 2 + 2 + 3). Interestingly, the grouping advantage increased with age and correlated with arithmetic abilities, with more math-skilled children showing stronger groupitizing effects. More recently, it has been reported that grouping by color can also decrease reaction times in adults^26^. Overall these studies suggest that serial counting without time constraints may be not a "pure" and direct measure of ANS precision, but could be tempered by arithmetical strategies, such as grouping, which involves processes such as parse-and-add.

In this study, we ask whether grouping items by spatial proximity or color not only increases enumeration speed but also increases precision (measured as Coefficient of variation). We also investigated whether this grouping phenomenon is a general property of numerosity perception, applying to temporal sequences as well as spatial arrays. The results suggest that groupitizing occurs for estimation of both temporal and spatial dimensions of numerosity. We also observed a robust inter-individual variability in the magnitude of grouping-based improvement, with participants who were less precise in estimating numerosity in random arrays benefiting more from the groupitizing. This suggests that some participants may take advantage of intrinsic grouping in random arrays to increase their performance, therefore benefit less from the explicit experimentally induced grouping.

## Methods

### Participants

Sixteen young adults (mean age = 26, standard deviation = 3.2, range = 23–36) participated in this study (12 male, 4 female, 13 participants were master’s students in psychology, 2 were grad-students and 1 a post-doc in neuroscience). All participants had normal or corrected-to-normal vision. All completed all tasks except one, who was unavailable to perform the two sequential numerosity conditions.

### Materials and procedure

Stimuli were created with Psychophysics toolbox for Matlab and displayed on a 60 Hz—15″ screen monitor (MacBook Pro) placed at viewing distance of 57 cm. Subjects were tested in a quite, dimly light room. The experimental procedures were approved by the local ethic committee (*Comitato Etico Pediatrico Regionale—Azienda Ospedaliero-Universitaria Meyer—Firenze FI*). The research was performed in accordance with Declaration of Helsinki and informed consent was obtained from all participants prior to each experiment.

**Figure 1:**
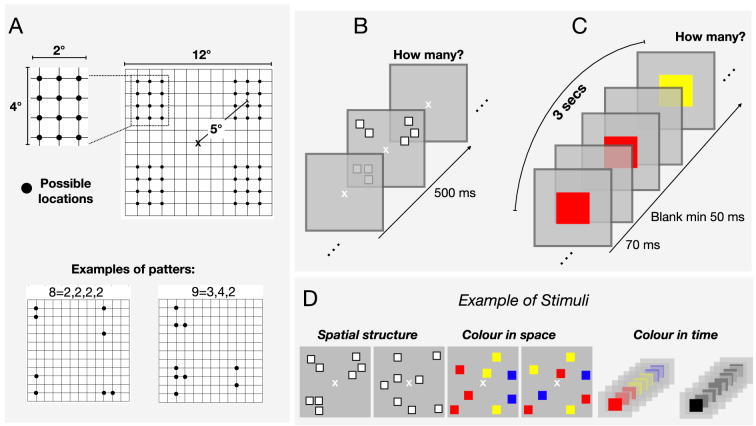
Stimuli and procedure. (**A**) Illustration about how stimulus position was defined in the grouping conditions (upper panel) with example configurations for numerosities 8 and 9 (lower panel). (**B**) Examples of stimuli arrangement in the various conditions, when grouping was defined by spatial proximity, color or temporal proximity, together with related random conditions (on the left-hand side). (**C**, **D**) Example of the time course for the spatial (**C**) and temporal (**D**) version of the experiment (tested in separate sessions). In the spatial numerosity conditions (**C**), a central patch of squares was presented for 500 ms. In the sequential numerosity condition (**D**) a series of squares was centrally presented. Participants were asked to verbally report the perceived numerosity. Stimuli are not depicted to scale.

Each trial started with a central fixation point that remained on screen for the entire experiment. After 500 ms a stimulus was displayed, followed by a blank screen. Participants estimated verbally the numerosity of the squares-array or square-sequence (in separate sessions with order pseudorandomized between subjects Fig. 1C, D).

The experimenter hit the spacebar when the participant responded (used to calculate reaction times), then entered the response on the numeric keypad, which initiated the following trial. Response time was measured from the stimulus offset to the beginning of vocalization. Participants were asked to respond as soon as possible, but also to concentrate on accuracy. Each condition was tested in separate blocks, and participants were never explicitly informed about the grouping cues.

Numerosity levels ranged from 4 to 16 (grain of 1, resulting in 13 numerosity levels). In the structured conditions, each numerosity was organized into clusters (between 2 and 4), each containing a variable number items (between 2 and 6), resulting in the following configurations: 2, 2−2, 2, 1−3, 3−2, 2, 2−3, 3, 1−3, 3, 2−2, 2, 2, 2−4, 4−4, 3, 2−4, 4, 1−3, 3, 3−3, 3, 3, 1−4, 4, 2, 1−3, 3, 3, 2−3, 3, 3, 3−4, 4, 4−4, 4, 3, 3−4, 4, 4, 3−5, 5, 3−4, 4, 4, 4−5, 5, 6. As numerosities 4 and 16 were not analyzed (see data analyses), each grouped pattern comprised a minimum of 2 and a maximum of 4 clusters. All clusters except one (13 = 5, 5, 3) contained from 1 to 4 elements. On each trial, a given numerosity and configuration pattern were randomly selected. Each participant completed about 150 trials for each of the six conditions (around 14,000 trials in total).

### Stimuli

#### Spatial arrays

Stimuli were arrays of squares (0.4° × 0.4°) displayed for 500 ms on each trial. Squares could not overlap and were constrained to fall within a 12°X12° virtual square area. In the conditions where spatial structure was manipulated, the individual items were white squares within black borders (so luminance was not a cue to number). In the unstructured conditions, the position of each square was randomly selected from 169 possible positions (within the stimulus area), being the centers of equally spread sectors within the 12°X12° area (each grid 1°X1°). For the spatially grouped condition, stimuli were arranged in 4 possible groups of 12 possible positions (see Fig. 1A). Each group (spanning over a max area of 4°X2°) was located in one quadrant and centered at 5° from the central fixation point. Each group was first randomly assigned to one quadrant (between 1 and 4), then the individual items positions was randomly selected between one of the 12 in the selected quadrant. Within each quadrant, the maximum center-to-center distance between each element was 4° and the minimum was 1°.

In the conditions where groups were defined by color, individual items could be red, green, blue or yellow (RGB: 255 0 0; 0 255 0; 0 0 255; 255 255 0). Color was assigned from left to right, so that similar colors appeared in vertical rows. For example, in the 3, 3, 2 condition depicted in Fig. 1B squares were first randomly located, then the first three squares (from the left border) were colored red, the next three yellow and the remaining two blue (colors randomly chosen for each group). In the unstructured color condition, positions were assigned with the same logic, but with colors assigned at random.

#### Temporal sequences

Stimuli were streams of 3° × 3° squares each presented at screen center for 70 ms, for a total trial duration of 3 s (Fig. 1D). The end of each trial was signaled by color change of the central fixation point, from white to green. Sequences were spaced pseudo-randomly: on every trial, a given number of impulses (chosen at random) were evenly spread within the 3-s sequence duration; then the timing of each impulse was randomly jittered by either ± 0, ± 20 or ± 40 ms to create a pseudorandom sequence of impulses with a minimum ISI between consecutive flashes of 50 ms. In the random condition all stimuli were black, while in the grouped condition they were grouped by color: each flash within a group could be red, green, blue or yellow (color coordinates as before), with group color randomly assigned. For example, in the 3, 3, 2 condition depicted in Fig. 1B, the first three flashes were colored red, the following were yellow and the remaining two blue.

### Data analysis

Since participants were explicitly informed about the numerical range (4–16), we eliminated the two extreme numerosities from the analyses. We controlled for response outliers by eliminating trials with RTs longer than 3 standard deviations from the average response time, calculated separately for each numerosity level and participant.

For each participant, we calculated for each numerosity the average perceived numerosity, the standard deviation of the responses and the median response time. Precision was measured by normalizing the standard deviation by the physical numerosity yielding the Coefficient of variation (CV), a dimensionless index of precision that allows comparison and averaging of performance across numerosities.

1 $$CV= \frac{{}_{i}}{{N}_{i}}$$

where ${N}_{i}$ is the analyzed numerosity and ${}_{i}$ the standard deviation of responses to numerosity *i*. Improvement (I) by grouping was measured by a normalized index yielding the proportion improvement:

2 $$I= \frac{{CV}_{R}-{CV}_{G}}{{CV}_{R}}$$

where ${CV}_{R}$ and ${CV}_{G}$ are the coefficient of variation for the random and grouped conditions.

Data were analyzed by repeated measures ANOVAs, and effect sizes were reported as η^2^, using JASP and Matlab.

## Results

We asked participants to estimate the numerosity of briefly presented visual impulses, presented either in simultaneous spatial arrays or temporal sequences. For both conditions (tested in separate sessions), we investigated the effects of task-irrelevant grouping cues on numerosity estimation precision and speed. Grouping manipulations mainly followed the formal definition of Starkey and McCandliss^25^ with both the number of groups and the number of items/events within each group falling within the subitizing range: 2, 3 or 4 groups each containing 1, 2, 3 or 4 items/events.

### Effect of grouping on perceived numerosity

**Figure 2:**
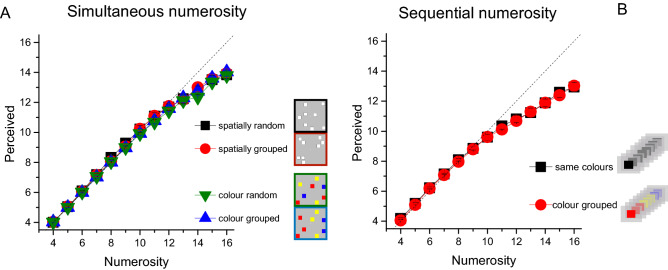
Perceived numerosity. Average perceived numerosity for spatial (**A**) and temporal (**B**) numerosity tasks, averaged across participants.

We first evaluated the effect of grouping on the accuracy of estimation of perceived numerosity. Figure 2 shows averaged responses as a function of physical numerosity. To statistically test differences across conditions, we ran Repeated measure ANOVAs (one for each numerosity format: simultaneous and sequential) with numerosity (11 levels, from N5 to N15) and grouping condition (4 or 2 levels for simultaneous and sequential numerosity respectively) as within subject factors. For both numerosity formats, the main effect of numerosity was obviously significant (simultaneous: F_(10,150)_ = 834.289, p < 0.001, η^2^ = 0.982; sequential: F_(10,140)_ = 282.289, p < 0.001, η^2^ = 0.953), but there was no significant effect of “condition” (simultaneous: F_(3,45)_ = 1.285, p = 0.29, η^2^ = 0.08; sequential: F_(1,14)_ = 0.281, p = 0.60, η^2^ = 0.02) and the condition-by-numerosity interactions were insignificant (simultaneous: F_(30,450)_ = 0.742, p = 0.84, η^2^ = 0.047; sequential: F_(10,140)_ = 0.311, p = 0.97, η^2^ = 0.022). Overall, these results clearly indicate that grouping did not significantly affect average perceived numerosity.

### Grouping and sensory precision

**Figure 3:**
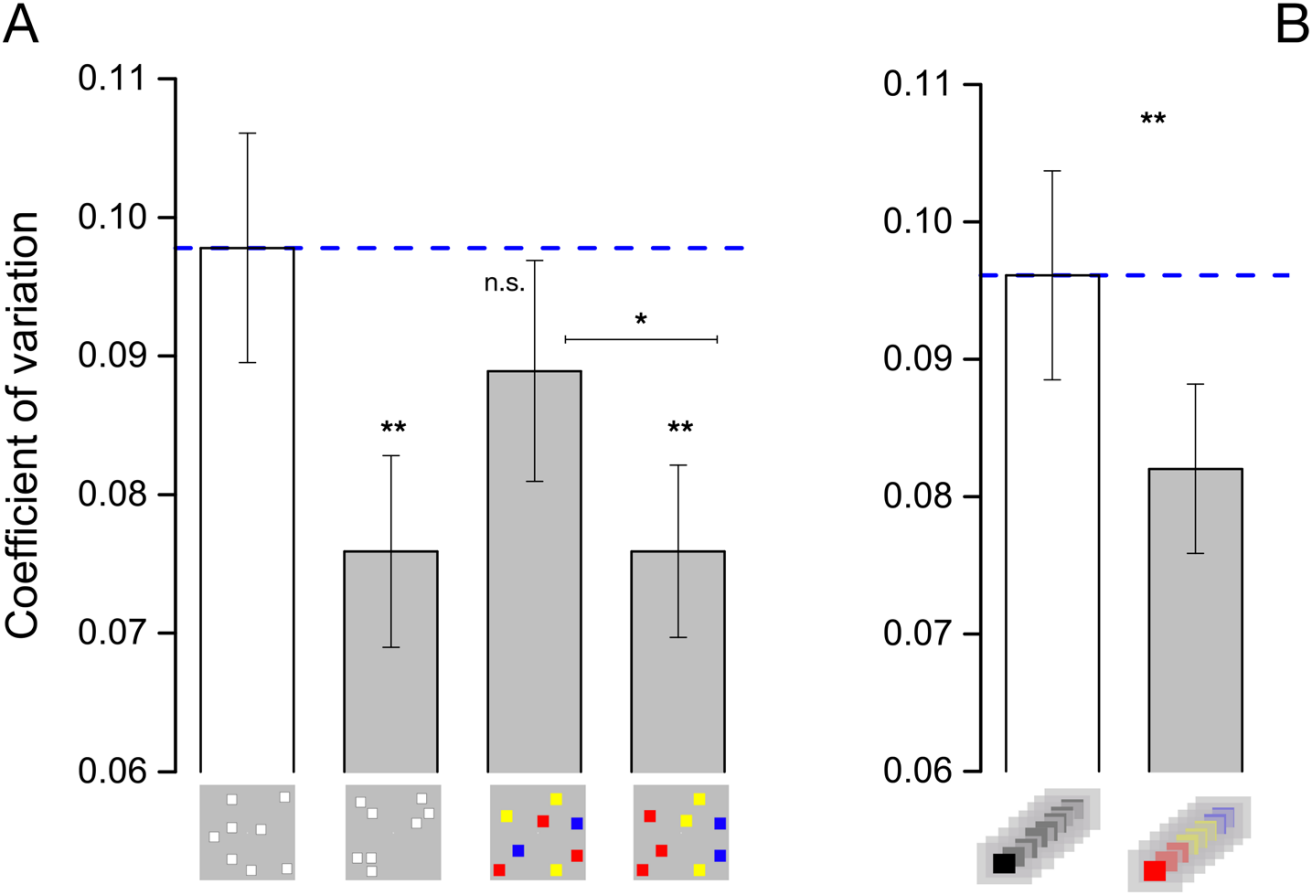
Sensory precision. Average Coefficient of variation for the simultaneous (**A**) and sequential (**B**) numerosity tasks. Error bar represent ± 1 s.e.m. **p ≤ 0.01 *p < 0.05.

Having established that grouping did not change average perceived numerosity (accuracy), we investigated its effect on sensory precision, indexed by Coefficient of variation (Eq. 1). This is a classical psychophysical parameter and, in the case of numerosity, is believed to reflect the sensory noise associated with the estimation process: higher values reflect less precision in the estimates and thus more sensory noise. Figure 3 shows Coefficient of variations averaged across numerosities and participants for the random and grouped conditions, for estimations of spatial (A) and temporal (B) numerosity.

For spatial presentations, Coefficient of variation was highest for the non-grouped condition, higher than all the grouped conditions. Repeated measures ANOVA with numerosity (11 levels) and condition (4 levels) revealed a significant main effect of condition (F_(3,45)_ = 4.9, p = 0.005, η^2^ = 0.247), with grouping decreasing Coefficient of variation compared to the spatially random condition (Fig. 3A). The effect of numerosity was also significant (F_(10,150)_ = 4.921, p < 0.001, η^2^ = 0.634), suggesting that Coefficient of variations are not constant with numerosity, while the interaction was not (F_(30,450)_ = 1.365, p = 0.097, η^2^ = 0.08), suggesting that the overall effect of grouping was constant across numerosity levels.

**Figure 4:**
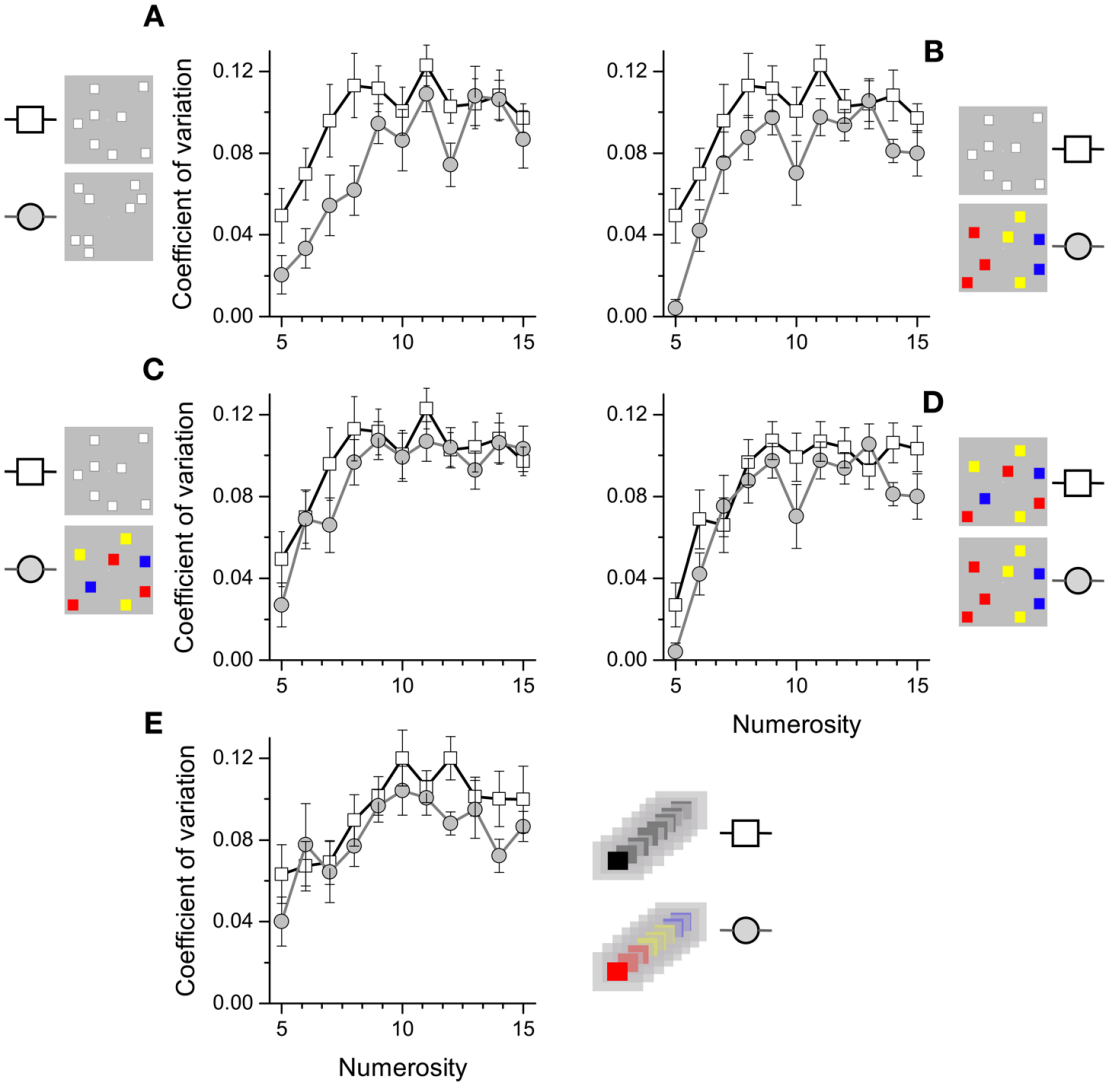
Sensory precision across numerosity levels. Average Coefficient of variation as a function of numerosity levels for all different experimental conditions. (**A**–**C**): Performance in the spatially random condition (open squares) against grouping by spatial proximity (**A**, gray circles), color gradient in space (**B**, gray circles) or only color (**C**, gray circles). (**D**) Coefficient of variations for the two-color conditions, random or grouped by color. (**E**) Coefficient of variation for the sequential presentation when stimuli were shared the same color (black) or similar colored items were presented temporally close to each other. Error bar represent ± 1 s.e.m.

To assess the effect of grouping separately for each condition, we then ran a series of repeated measures ANOVAs against the spatially random stimuli condition. The results revealed that grouping by spatial structure (F_(1,15)_ = 9.38, p = 0.008) and by color gradient in space (F_(1,15)_ = 13.908, p = 0.002) both induced a significant reduction of Coefficient of variation, and both had a quite large effect (spatial structure 22%, η^2^ = 0.43, color gradient in space 22%, η^2^ = 0.48). Grouping by color without spatial gradient did not produce a significant reduction in Coefficient of variation (9%, F_(1,15)_ = 2.264, p = 0.15, η^2^ = 0.13). The ANOVA comparing the two color conditions (with and without a spatial gradient) revealed that grouping by color with a gradient in space produced a significant reduction in Coefficient of variation compared to color alone (14% reduction in WF, F_(1,15)_ = 5.165, p = 0.038, η^2^ = 0.256). The interaction between numerosity-by-condition was never significant (p > 0.05) in any condition comparison (Fig. 4A–D), suggesting the effect was comparable across numerosity levels.

Figure 3B shows the effects of grouping on sequential numerosity. Here, grouping was encouraged with sequences of same-colored flashes within the sequence. Again, grouping yielded a clear increase in precision compared to the random condition, with a Coefficient of variation reduction of about 15% (F_(1,14)_ = 11.683, p = 0.004, η^2^ = 0.455). Once again, the numerosity-by-condition interaction was not significant (p > 0.05).

### Grouping and response times

**Figure 5:**
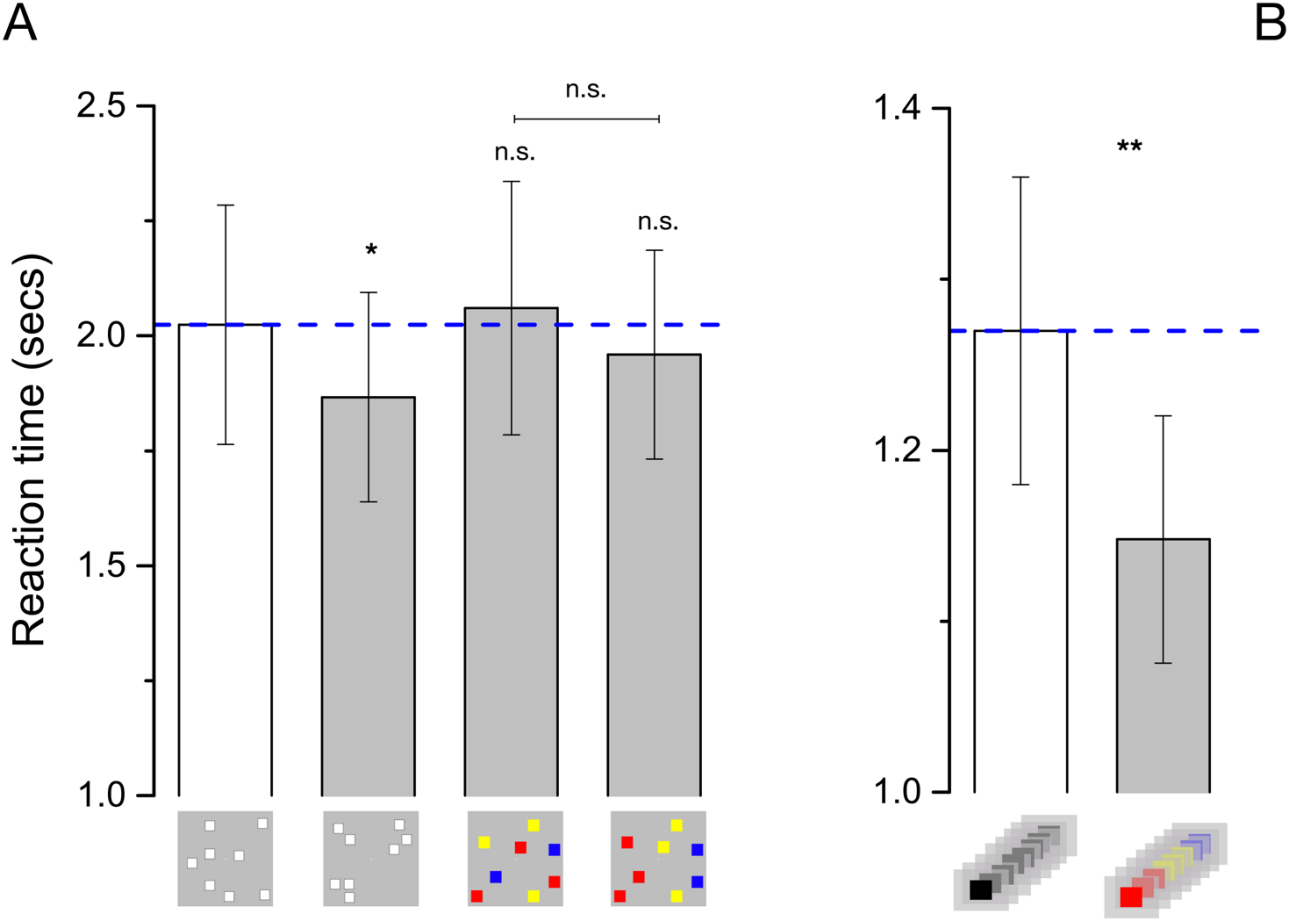
Reaction times. Average Reaction times for the various experimental conditions for simultaneous (**A**) and sequential numerosity (**B**) formats. Error bar represent ± 1 s.e.m. ***p < 0.001, **p < 0.01, *p < 0.05.

Like previous studies in the literature^25,26^, we also investigated the effect of grouping in term of response speed (Fig. 5). Reaction times were around 2 s for all experiments with spatial arrays (Fig. 5A), and around 1.2 s for the temporal sequences (Fig. 5B).

Repeated measure ANOVA with numerosity (11 levels) and condition (4 levels) as factors did not reveal a significant effect of spatial grouping condition (F_(3,45)_ = 1.008, p = 0.40, η^2^ = 0.06). However, separate repeated measure ANOVAs against spatially random stimuli revealed that grouping by spatial structure significantly reduced RTs from 2.02 ± 0.26 to 1.86 ± 0.22 s, an effect of 8% (F_(1,15)_ = 4.612, p = 0.048, η^2^ = 0.235, for all the other ANOVAs min p = 0.25). There was a significant reduction of response time induced by grouping of temporal sequences (RT unstructured = 1.27 ± 0.083, RT grouped = 1.14 ± 0.072, an effect of 10%: F_(1,14)_ = 8.861, p = 0.01, η^2^ = 0.388). Again, the effect of numerosity was statistically significant (F_(10,140)_ = 10.13, p < 0.001, η^2^ = 0.42) but not the numerosity-by-condition interaction (F_(10,140)_ = 0.924, p = 0.513, η^2^ = 0.062). Finally, all ANOVAs revealed a statistically significant effect of numerosity (reaction times increased with set size, all p < 0.001), but no numerosity-by-condition interactions (p > 0.05).

### Interindividual differences in grouping advantage

The results so far show that grouping stimuli into easily separable, subitizable chunks yielded more precise estimates than with random patterns. The effect is robust, but there is also considerable interindividual variability. Here we asked whether the magnitude of improvement may be related to the baseline sensory precision. It is feasible that some participants always use grouping strategies to some extent, taking advantage of the intrinsic clustering of random patterns. If this were the case, we would expect these participants to benefit less from explicit grouping, as they were already using this strategy. That is to say, participants with the highest Coefficient of variations measured in the ungrouped conditions should benefit the most from the explicit grouping.

**Figure 6:**
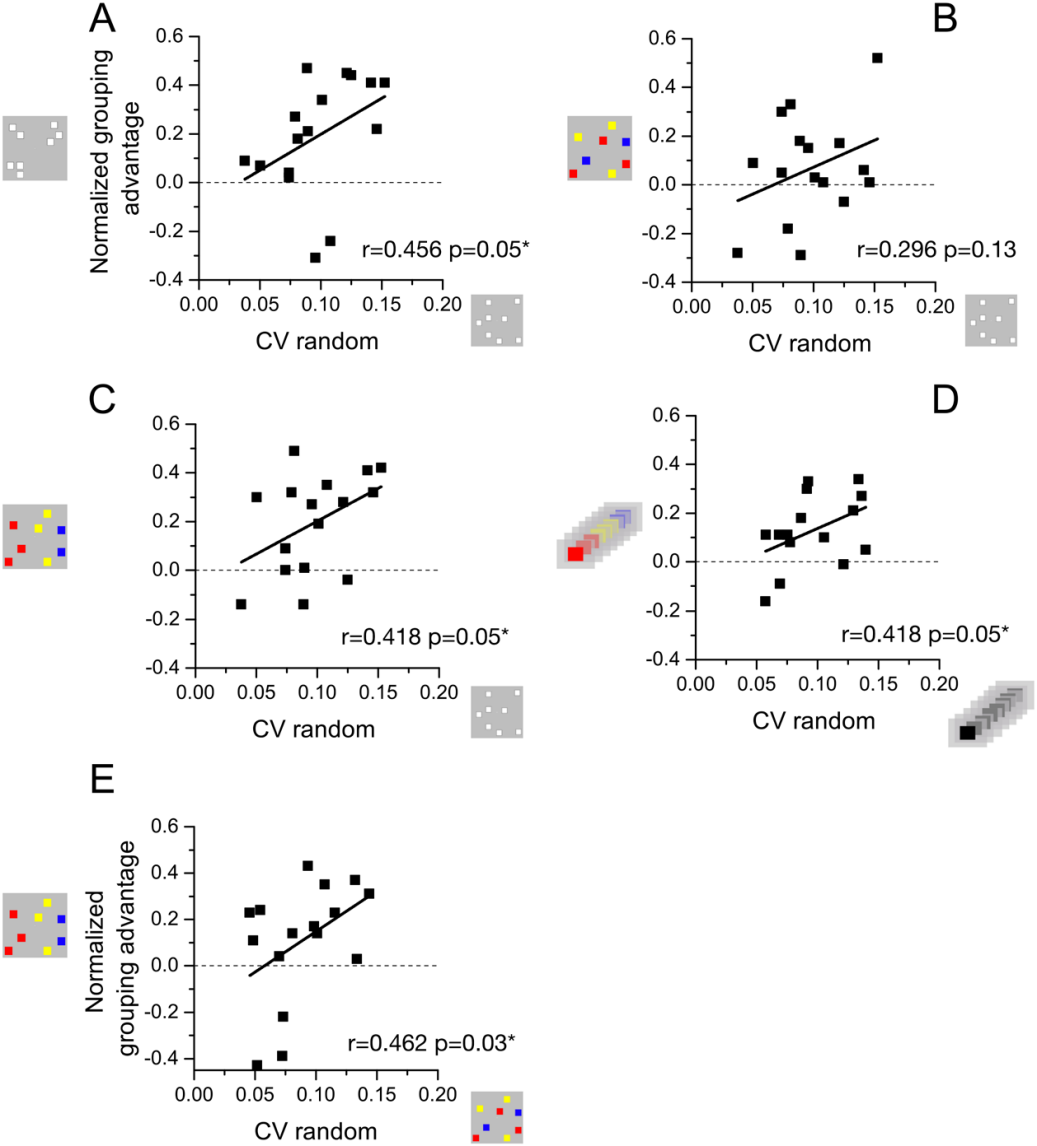
Individual differences. Correlations between grouping effects (normalized improvement by grouping) on estimation precision and Coefficient of variation in the different experimental conditions [(**A**): spatial grouping, (**B**, **C**, **E**): colour groping on spatial numerosity, (**D**) colour groping on sequential numerosity]. Positive values indicate a reduction of Coefficient of variation induced by grouping. Positive correlations (Pearson r) indicate that participants with worse sensory precision in the unstructured conditions (abscissa) gained more from grouping. Lines are best linear fit, one tailed p-values. *p ≤ 0.05.

To test this notion, we correlated the magnitude of the grouping advantage (the normalized improvement by grouping (Eq. 2) against the baseline Coefficient of variation (Fig. 6). If grouping were to reduce all Coefficient of variations proportionally (multiplicatively), the correlation should be zero. If the effect were additive, then the correlation would be negative (proportionally greater for the lower Coefficient of variations). However, if those who had the highest Coefficient of variations profited proportionally more than those with lower Coefficient of variations, the correlations should be positive.

For the four conditions that yielded a significant grouping effect – spatial grouping, color clustering (with and without spatial grouping) and temporal color clustering—the correlation was significantly positive (p < 0.05, one-tailed test). On the other hand, the condition in which grouping did not yield a significant advantage on numerosity precision (random space Vs random color in space), showed no significant advantage (p = 0.10).

## Discussion

This study shows that using color, or spatial or temporal proximity to group items together robustly improves the precision of numerosity estimation, by up to 20%.

The magnitude of the advantage for grouping did not vary with numerosity, over the range tested, from 5 to 15 (Fig. 4). That is interesting, as one may have expected proportionally greater effects for the larger numbers. But perhaps there was also a greater cost in subitizing and doing addition with larger numbers, so the net proportional gain was similar. We selected our number range to be comfortably inside the range where numbers are thought to be estimated directly, rather than via texture-density mechanisms^27,28^. It would be interesting to test much higher numerosities and densities, to see if grouping can also aid in judgments of texture density. It would seem unlikely if based on subitizing, as subitizing is limited to about 4, but worth verifying.

We also found smaller and less robust advantages in reaction times, confirming previous studies^25,26^. We found that grouping by spatial structure slightly reduced reaction times relative to the spatial random condition, by about 8%. However, RTs in the spatial gradient color condition were not significantly different from the random color condition. One possibility for this discrepancy may be that grouping by the spatial dimension is more salient compared with grouping by color. Alternatively, colored items may induce a strong tendency to automatically group the stimuli, even when randomly scattered spatially. This idea is supported by the lower CVs in the random condition with coloured stimuli compared to those measured with achromatic stimuli. Not surprisingly, this statistically insignificant trend was not evident in the RTs, in line with the fact that in the present study RTs have proven to be less robust in detecting grouping effects than CVs.

Previous research has shown that grouping, or *groupitizing*, speeds up serial counting^25^, but this does not help preschoolers. Furthermore, the grouping advantage correlated positively with arithmetical abilities in school-age children, suggesting that grouping relies, at least to some extent, on formal arithmetical knowledge. Thus grouping may reflect an implicit math strategy of numerosity perception, like “parse the scene into subitizable groups then sum the subitized estimates”. That grouping not only speeds counting but also lowers numerosity estimation thresholds has broad implications. Precision in numerosity estimation and discrimination are predictive of child math abilities^5,29^, and are both impaired in dyscalculia^6,10^. These results have been interpreted as a link between the perceptual ability to estimate numerosity and the cognitive ability to learn math^6,15^. However, if grouping strategies are spontaneously used by some participants, such as those with more spontaneous arithmetical skills, it could be this that mediates the link between numerosity and math proficiency. Use of grouping information, either intrinsic or explicitly introduced, requires some basic math skills, such as rapid addition of the numerosities of the sub-groups. It is likely that participants who opt for this strategy—rather than a global appraisal of the whole pattern—would be those with the greater math skills. This would have important implications for understanding the link between measures of numerosity sensitivity and math.

In the present study, participants who were more precise in the random condition benefited proportionately less from grouped configurations than those with higher thresholds. One plausible explanation for this is that those with lower thresholds use grouping strategies even with the random patterns, taking advantage of intrinsic grouping in randomness. These people may benefit less from the explicit grouping imposed by spatial or temporal proximity, and therefore show less improvement. This possibility is interesting, with implications about different individual styles in numerosity perception, well worth pursuing further.

The correlation between numerosity precision and math skills is interesting. While thresholds for estimating numerosities at moderate, uncrowded densities predict well math performance^5,6,8^, numerosity discriminations at high densities^7^ do not; nor does subitizing^13^. Furthermore, thresholds for temporal sequences do not predict math performance^29^, despite the clear evidence for a generalized number system encompassing space and time^28,30,31^. All this suggests that some aspect of estimation of numerosity at low densities is related to math. A clear candidate mechanism could be “groupitizing”, the use of strategic grouping to parse arrays into subitizable chunks. As mentioned above, this strategy requires some basic arithmetical skills: simple but rapid addition. It is reasonable to suppose that this skill does not help in the subitizing range, where arrays are already subitizable without further parsing, so that is not predictive of math. Similarly, for high numerosities the parsing strategy would not be effective, as only a limited number of subitizable sub-sets can be counted. Why estimation of temporal numerosity sequences does not correlate with math is less clear, as the present results show that a grouping strategy is possible with temporal sequences, and that those who benefit most from the grouping cues are those with highest thresholds. Perhaps the fact that temporal sequences are necessarily one-dimensional makes it harder to spontaneously group into sub-sets, particularly for young children. Also, in previous studies the presentations were constrained to be quite rhythmic, which does not lend to spontaneous parsing into groups. And perhaps phenomena such as “entrainment”^32,33^ tend to make the sequences even more rhythmical, and hard to group. Again, this idea bears further investigation, particularly with children.

To conclude, the current study demonstrated that use of grouping strategies can aid considerably in the estimation of numerosity. The strategy may be related to mathematical abilities, and understanding it better could be of considerable importance in understanding the link between estimating numerosity and formal math skills.
